# Estimation of Walking Speed and Its Spatiotemporal Determinants Using a Single Inertial Sensor Worn on the Thigh: From Healthy to Hemiparetic Walking

**DOI:** 10.3390/s21216976

**Published:** 2021-10-21

**Authors:** Dheepak Arumukhom Revi, Stefano M. M. De Rossi, Conor J. Walsh, Louis N. Awad

**Affiliations:** 1 Neuromotor Recovery Laboratory, Department of Physical Therapy and Athletic Training, College of Health and Rehabilitation Sciences: Sargent College, Boston University, Boston, MA 02215, USA; dheepak1@bu.edu (D.A.R.); sderossi@bu.edu (S.M.M.D.R.); 2 Department of Mechanical Engineering, Boston University, Boston, MA 02215, USA; 3 John A. Paulson School of Engineering and Applied Sciences, Harvard University, Cambridge, MA 02138, USA; walsh@seas.harvard.edu

**Keywords:** walking speed estimation, wearable sensors, thigh, phase portrait

## Abstract

We present the use of a single inertial measurement unit (IMU) worn on the thigh to produce stride-by-stride estimates of walking speed and its spatiotemporal determinants (i.e., stride time and stride length). Ten healthy and eight post-stroke individuals completed a 6-min walk test with an 18-camera motion capture system used for ground truth measurements. Subject-specific estimation models were trained to estimate walking speed using the polar radius extracted from phase portraits produced from the IMU-measured thigh angular position and velocity. Consecutive flexion peaks in the thigh angular position data were used to define each stride and compute stride times. Stride-by-stride estimates of walking speed and stride time were then used to compute stride length. In both the healthy and post-stroke cohorts, low error and high consistency were observed for the IMU estimates of walking speed (MAE < 0.035 m/s; ICC > 0.98), stride time (MAE < 30 ms; ICC > 0.97), and stride length (MAE < 0.037 m; ICC > 0.96). This study advances the use of a single wearable sensor to accurately estimate walking speed and its spatiotemporal determinants during both healthy and hemiparetic walking.

## 1. Introduction

People post-stroke indicate that deficits in how far and fast they walk limit their ability to engage in home and community activities [1]. Indeed, the total distance that a person can walk during the 6-min walk test—a popular, clinic-based test of long-distance walking capacity—has been shown to predict community participation and reintegration after stroke [2,3]. More recently, the distance-induced change in walking speed during the 6-min walk test has been used to classify people post-stroke into endurant versus non-endurant subgroups, with the non-endurant group presenting with substantially less everyday community walking activity [4]. That is, regardless of how far they can walk during the 6 minute walk test, individuals who slow down during the test walk less in the community than more endurant individuals. Beyond long-distance walking speed, real-world community walking activity is also associated with step-to-step variability in the spatiotemporal determinants of walking speed (i.e., stride length and stride time); individuals with higher gait variability have lower balance confidence, increased fall rates, and less physical activity [5,6,7,8,9].

The advance of wearable sensors and digital assessment tools that can track salient walking metrics during everyday walking activities is of high clinical significance given the importance of walking activity to health and quality of life [10,11]. Current approaches to measuring walking speed and its spatiotemporal determinants consist of, on one hand, highly precise measurements made with expensive, laboratory-based motion analysis instrumentation [11,12] versus, on the other hand, less precise clinic-based approaches centered on visual gait analysis and patient self-report [11,13,14,15,16]. More recently, portable inertial measurement units (IMUs) have shown the potential to overcome the physical constraints of traditional motion capture laboratories [11,14,16,17], allowing objective walking assessments beyond the lab. Indeed, IMUs have the potential to collect data continuously in the background of everyday walking activities; these data can advance new healthcare paradigms for aging adults and people with chronic conditions [18,19]. For example, because gait changes may precede a change in health status [20], the detection of gait changes during long-term gait monitoring can trigger the deployment of interventions to mitigate functional decline and prevent catastrophic events, such as a fall.

In the last two decades, several methods for IMU-based estimation of walking speed and its spatiotemporal determinants have emerged [14,21,22,23,24,25,26,27,28,29]. Many of these approaches exploit gait features that, though readily found in the walking patterns of healthy individuals, vary widely across patient populations. Indeed, stereotypical gait events—like heel strike, foot flat, shank vertical, and others [21,23,24]—are important for these approaches, but are difficult to detect in the face of highly variable and impaired gait kinematics, such as found in people post-stroke. As a result, estimation approaches that work well for individuals with typical gait patterns cannot be assumed to have similar accuracy when applied to individuals with atypical gait patterns [14,18,24,27]. For example, Laudanski et al. showed that a single IMU worn on the foot could be used to estimate walking speed with a root mean square error of 0.09 m/s [24], but when the same approach was tested in the same individuals walking with externally rotated feet (i.e., toe-out gait, which is commonly present in patient populations [30,31]), a higher error of 0.23 m/s was observed [24]. The magnitude of error was lower when estimates were made using a more proximally-mounted shank IMU (i.e., 0.06 m/s), but even with a shank IMU a substantial increase in error was observed in the toe-out gait condition (i.e., 0.10 m/s) [24].

Among people with post-stroke hemiparesis, the use of multiple IMUs located across the paretic and non-paretic limbs has proven effective in improving estimation accuracy [28]. However, increasing the number of sensors poses usability challenges, especially when these sensors must be worn distally on the shank or foot. Indeed, for individuals with limited range of motion due to musculoskeletal [32,33] or neurological [34] conditions, IMUs worn proximally at the waist or on the thigh may be easier to self-manage than distally-worn IMUs. Relatedly, a thigh-worn IMU may be more desirable for embedded control of wearable robots and orthoses that do not extend below the knee [23,35,36]. Beyond usability, thigh-worn IMUs may be preferable to distally-worn IMUs from a measurement perspective. Indeed, in addition to potentially being less susceptible to errors arising from out-of-plane movements by lower segments (as described in [24]), the proximo-distal hypothesis of joint coordination during walking suggests that changes in the walking pattern emerge largely from feed-forward control of the proximal leg muscles [37], and thigh data collected during walking have been shown to be highly predictive of distal joint motion [38].

In this paper, we describe a new approach to using a single inertial sensor worn on the thigh to estimate, on a stride-by-stride basis, overground walking speed and its spatio-temporal determinants. More specifically, measurements of angular position and velocity made by a thigh-worn IMU are used to generate phase portraits (i.e., time-independent orbital representations of movement). Phase-portrait metrics are then used as inputs in subject-specific estimation models. The accuracy of this estimation approach is eval-uated using long-distance walking data collected on both young healthy individuals and individuals with post-stroke hemiparesis.

## 2. Materials and Methods

### 2.1. Participants

Ten healthy individuals (26 ± 4 years, 171 ± 11 cm, 68 ± 17 kg) (Table 1, top) and eight individuals in the chronic phase after stroke (61 ± 12 years, 178 ± 4 cm, 88 ± 10 kg) (Table 1, bottom) participated in the study. Post-stroke study participants could walk without the assistance of another individual but showed observable gait deficits. Exclusion criteria included conditions other than stroke that impaired walking, an inability to communicate with the study team members, and pain during walking. All study procedures were approved by the Institutional Review Board at Boston University and written informed consent was obtained from all study participants.

### 2.2. Gait Evaluation and Data Collection

To begin each testing session, study participants completed a standing static trial, a 10-m walk test at a comfortable walking speed, and a 6-min walk test [3]. The standing static trial served as a reference for the orientation of the IMUs, the 10-m walk test was used to characterize individuals based on their baseline comfortable walking speed (see Table 1), and the 6-min walk test provided the training and validation data used in the study.

**Figure 1:**
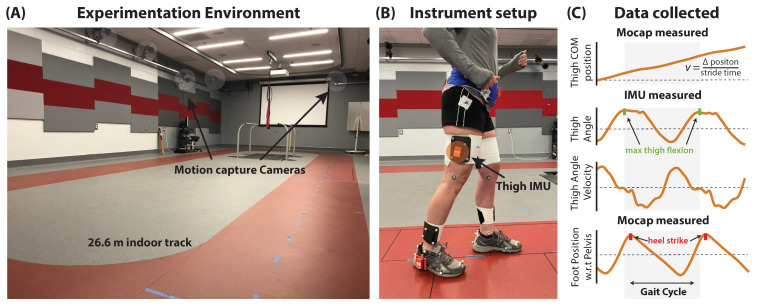
Study Overview: (**A**) Optical motion capture provided ground truth measurements. (**B**) A thigh inertial measurement unit (IMU) provided all data used for the IMU-based estimation. (**C**) Example motion capture (Mocap) and IMU measured data collected and used in the study. Abbreviations: IMU—inertial measurement unit, COM—center of mass; w.r.t—with respect to.

Data were collected using wireless inertial measurement units (IMUs, MTw Awinda, Xsens, Enschede, Netherlands; 100 Hz) attached securely over marker clusters located laterally on both thighs. Their position was a third of the thigh segment’s length above the knee joint (Figure 1B). The thigh IMU-marker cluster was positioned such that one axis always moved along the sagittal plane. Though IMU data were collected from both limbs, all estimates were made using IMU data collected only from the limb of interest. The IMU and motion capture signals were time-synchronized using a synchronization pulse triggered at the start of data collection [39]. All walking tests were performed around a 26.6 m oval indoor track consisting of two 10 m straightaways separated by 3.3 m turns on either end. One of the 10 m straightaways included the capture area for the 18-camera motion capture system (Qualisys, Göteborg, Sweden; 200 Hz) that provided the study’s ground truth reference data (Figure 1A).

Only strides within the available motion capture region could be used for comparative analyses. That is, although IMU-based estimates are available for all strides, because the motion capture region included only one 10 m straightaway, strides during turns and the other straightaway were excluded. To identify turns, during periods of walking along the known straightaways, the average change within a stride in the Yaw angle (i.e., rotation along the transverse plane) was measured by the thigh IMU. A threshold set to three standard deviations from this average was used to identify turns.

### 2.3. Data Processing

Raw IMU data (i.e., raw acceleration, gyro and quaternion angles) were collected using XSens MT Manager at 100 Hz and filtered at 10 Hz using a second-order Butterworth filter. Motion capture data were collected at 200 Hz, downsampled to match the IMU collection frequency, and filtered at 10 Hz using a second-order Butterworth filter. The IMU orientations (i.e. Euler angles) during the static standing trial served as the zero reference for the IMU orientation signals during walking [17,40,41]. To compute the segment Euler angles, the quaternion vector relative to the zero reference was found and subsequently rotated such that the roll axis of the IMU was aligned with the sagittal rotation axis of the segment [17,21,42,43].

### 2.4. Analysis

Stride-by-stride estimates of walking speed and its spatiotemporal determinants require segmenting time series data into individual strides. Commonly, consecutive heel strikes by the same foot are used to define a single stride [12,44]; however, any gait event that repeats across strides may be used [12,26]. Our *first* analysis goal was to determine the validity of defining a stride using repeating gait events observed in the thigh angle data directly measured by the thigh IMU—specifically, repeating maximum thigh flexion events—and using this definition to compute stride times (Section 2.4.1). Our *second* analysis goal was to evaluate the accuracy of a phase-portrait-based approach to estimating, on a stride-by-stride basis, walking speed and stride length (Section 2.4.2). Phase portraits have been used in a wide range of movement analysis applications, including the study of balance [11,45], walking detection [11,26], and prosthesis control [46]. In the estimation approach presented in this paper, subject-specific models derive estimates of walking speed using the polar radius computed from the phase portrait produced by coupling the thigh angle and its first derivative (i.e., angular velocity). Our *third* analysis goal was to examine clinical applications of these estimates, including the utility of a potential movement biomarker extracted from the thigh phase portrait (i.e., its roundness) (Section 2.4.3 and Section 2.4.4).

#### 2.4.1. Stride Time Measurement Using a Thigh IMU

IMU-based measurements of stride time ($ST_{est}$) were compared to ground truth motion capture measurements ($ST_{act}$) (Figure 1C). $ST_{act}$ was computed as follows: (i) The position of the foot with respect to the pelvis was tracked using segment clusters. (ii) The peaks of the resulting sinusoidal curve were used to identify heel strike gait events [44]. (iii) The time difference between consecutive heel strikes by the same limb was defined as $ST_{act}$. $ST_{est}$ was computed as follows: (i) The thigh angle was tracked by a thigh IMU. (ii) The peaks in the IMU-measured thigh angle were used to identify maximum thigh flexion gait events [26,36,38]. (iii) The time difference between consecutive maximum thigh flexion events defined $ST_{est}$. Given strong agreement between $ST_{act}$ and $ST_{est}$ (see Section 3.1), IMU-based segmentation was used for all analyses, with each stride time-normalized to 100 points.

#### 2.4.2. Walking Speed and Stride Length Estimation Using a Thigh IMU

**Figure 2:**
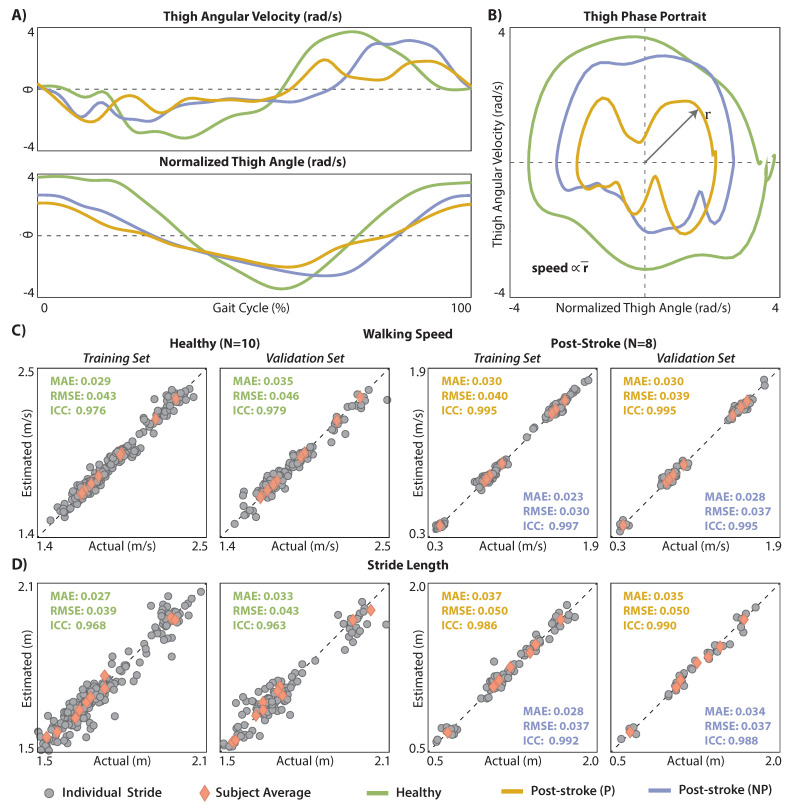
Thigh phase portrait and walking speed and stride length estimation accuracy: (**A**) Angular position and velocity measured by a thigh IMU (after filtering and normalization of the thigh angle) for one healthy study participant and the paretic and nonparetic limbs of a person post-stroke. (**B**) Exemplar phase portraits generated using data plotted in (**A**). (**C**) Accuracy of walking speed estimation within and across subjects. (**D**) Accuracy of stride length estimation within and across subjects.

IMU-based estimates of walking speed ($v_{est}$) were compared to ground truth motion capture measurements ($v_{act}$). $v_{act}$ was computed, on a stride-by-stride basis, as follows: (i) Thigh position was tracked using the thigh marker cluster, (ii) differentiated to yield instantaneous thigh velocity, (iii) which was then segmented between consecutive maximum thigh flexion events, and (iv) averaged. Our IMU-based approach to computing $v_{est}$ builds on promising preliminary work by Quintero et al. that reported, for one healthy individual walking on a treadmill, a strong linear relationship between treadmill walking speed and the polar radius extracted from phase portraits of the thigh angle ($\theta_{x}$) and its first derivative ($\dot{\theta_{y}}$) in the sagittal plane (see [26,46]). More specifically, as described in [26,47], to produce a thigh phase portrait, $\theta_{x}$ and $\dot{\theta_{y}}$ are plotted against each other, with the origin shifted to (0,0) and $\theta_{x}$ scaled using the maximum and minimum values of $\dot{\theta_{y}}$—that is $\theta_{x}$ is represented as rad/s (Figure 2A,B). In their prior work, Quintero et al. used an interpolating filter to smooth the IMU data and then computed the average polar radius ($\bar{r}$) from the fourth quadrant of the phase portrait as the vector norm between $\theta_{x}$ and $\dot{\theta_{y}}$ (Equation (1)).

(1) $$r=\sqrt{{\left(\theta_{x}\right)}^{2}+{\left(\dot{\theta_{y}}\right)}^{2}}$$

To adapt this approach for post-stroke gait estimation during overground walking, we (i) did not apply the interpolating filter to the IMU data and (ii) computed the average polar radius across all quadrants, instead of just the fourth quadrant. The interpolating filter introduced by Quintero et al. aims to remove unwanted disturbances in the IMU data (e.g., due to heel strike impacts), which may be necessary for real-time robotic control applications. To minimize filtering out real kinematic features arising from post-stroke gait impairment that may be important for gait parameter estimation, we used a more conventional, second-order Butterworth filter. Moreover, to increase estimation robustness in patient populations with heterogeneous gait deficits that may differentially affect the average polar radius computed from any one quadrant, we used the average polar radius across all four quadrants instead of the fourth quadrant’s polar radius. A comparative analysis of phase portrait metrics and walking speed estimates without and with these changes is presented in Appendix A.

Subject-specific models were trained to exploit the strong linear relationship between IMU-derived estimates of $\bar{r}$ and $v_{act}$. More specifically, using the training dataset (see Section 2.4.5), Equation (2) was used to identify subject-specific coefficients ($c_{1},c_{2}$) that best relate $\bar{r}$ and $v_{act}$. Once identified, the subject-specific coefficients can then be used together with $\bar{r}$ to produce $v_{est}$. $v_{est}$ was computed for both the training and validation datasets.

(2) $$v=c_{1}\cdot \bar{r}+c_{2}$$

Given $v_{est}$ and $ST_{est}$, IMU-based estimates of stride length ($SL_{est}$) were computed using Equation (3). Ground truth measurements of stride length ($SL_{act}$) were also computed using Equation (3), using $v_{act}$ instead of $v_{est}$.

(3) SL=v×ST

#### 2.4.3. Exploratory Application 1: Distance-Induced Changes in Speed and Its Determinants

As an exploratory clinical application of our IMU-based gait estimation approach, we plotted the distance-induced changes in $v_{est}$, $ST_{est}$, and $SL_{est}$ to visualize subject-specific changes during both healthy and post-stroke walking. To facilitate clinical interpretation, $ST_{est}$ is reported as cadence (i.e., $Cadence=120/ST_{est}$).

#### 2.4.4. Exploratory Application 2: Phase Portrait Roundness as a Movement Biomarker

A dominant feature of the phase portrait is its circular orbit [26]. A second exploratory clinical application of our IMU-based gait estimation approach was to examine if the “roundness” of the phase portraits produced by the thigh IMU data collected during long-distance walking differed between healthy walking and post-stroke walking, and between the paretic and non-paretic limbs of people post-stroke. We quantified roundness as the ratio between the inscribed and the circumscribed circle; that is, the ratio between the largest circle that fits inside the phase portrait and the smallest circle that can enclose the phase portrait [48]. Based on this definition, a perfect circle would have a roundness of 1; a roundness of less than 1 indicates a deviation from the perfect circular orbit. Although thigh phase portraits generated from healthy walking data are not expected to have perfect roundness of 1, we expect gait deficits that impair the thigh angle, velocity, or coordination of angle and velocity to result in deviations in the circular orbit of the phase portrait and decrease the roundness measurement.

#### 2.4.5. Statistical Analysis

Two-thirds of each subject’s 6-min walk test data were used for model training; the remaining one-third was used for validation. More specifically, to take advantage of the natural gait variability present over the duration of the 6-min walk test, two out of every three strides were used for training and the third stride was used for validation. Estimation accuracy is reported for both training and validation datasets in the figures, and for just the validation dataset in the text of Section 3.2.

Subject-specific linear models were fit using the fitlm() function, with robust fit “on” to minimize the effects of outliers. To assess the accuracy of the IMU-based estimates ($v_{est}$, $ST_{est}$, and $SL_{est}$) versus the ground truth motion capture measurements ($v_{act}$, $ST_{act}$, and $SL_{act}$), mean absolute error (MAE) and root mean square error (RMSE) were computed. In addition, the degree of absolute agreement among the estimated and actual measurements was evaluated using two-way mixed effect, absolute agreement, single rater intraclass correlation coefficients (ICCs) [49,50]. ICC values above 0.9 were considered to be excellent, between 0.9 and 0.75 as good, and between 0.75 and 0.5 as moderate [49].

**Figure 3:**
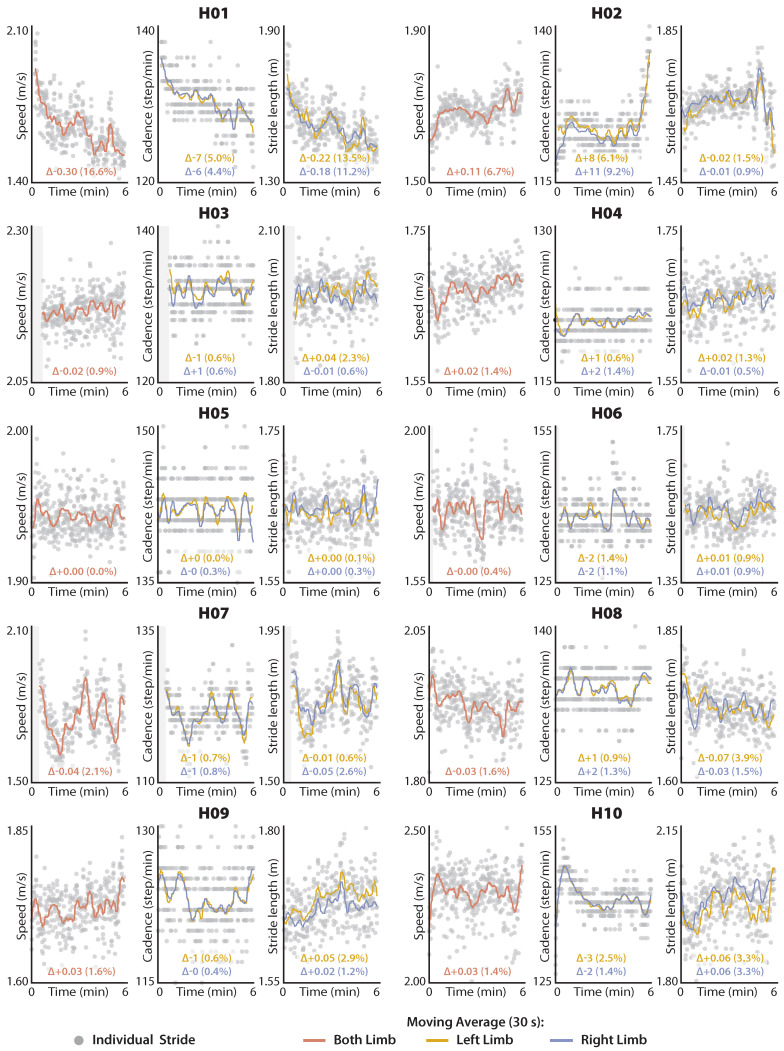
Distance-induced changes in speed, stride length, and cadence in healthy individuals. Reported Δs are the difference in the average from the last 30 s of walking and the first 30 s of walking. Regions highlighted in gray are missing data.

**Figure 4:**
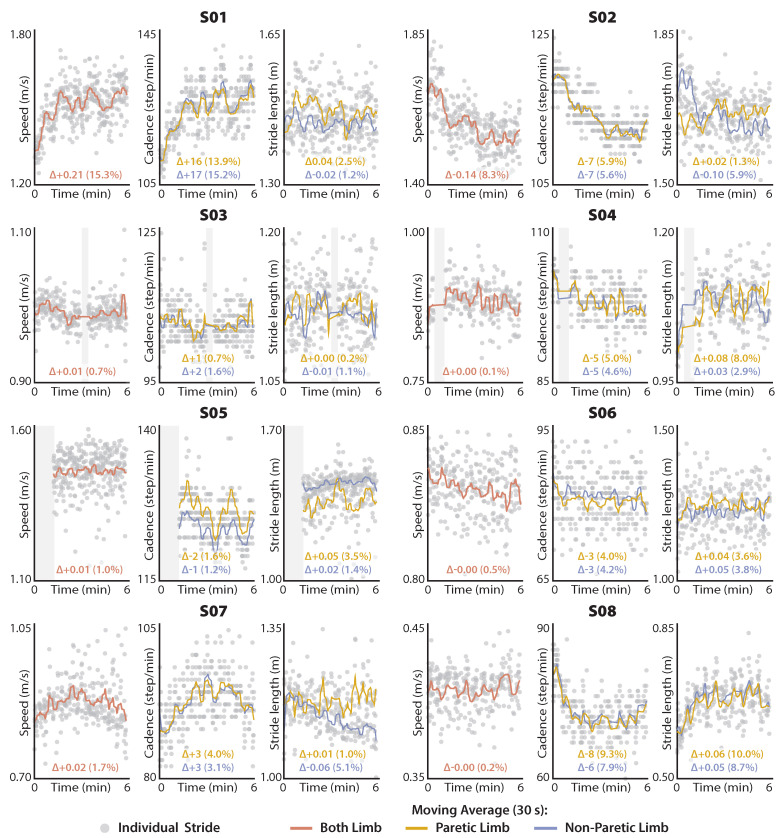
Distance-induced changes in speed, stride length, and cadence in post-stroke individuals. Reported Δs are the difference in the average from the last 30 s of walking and the first 30 s of walking. Regions highlighted in gray are missing data.

The comparative analyses of ground truth motion capture measurements versus IMU-based estimates are inherently limited to the strides with available motion capture data. In contrast, because the two exploratory clinical evaluations—i.e., (i) visualizing distance-induced changes in walking speed and its determinants and (ii) evaluating phase-portrait roundness as a movement biomarker—require only IMU data, all available strides are used. For the first exploratory clinical evaluation (Section 2.4.3), a 30 s Gaussian-weighted moving average filter was applied to the stride-by-stride estimates of $v_{est}$, $ST_{est}$, and $SL_{est}$ using the smoothdata() function and presented alongside the individual stride data in Figure 3 and Figure 4. For the second exploratory clinical evaluation (Section 2.4.4), Wilcoxon Rank Sum tests were used to evaluate differences in thigh phase portrait roundness across healthy, paretic, and nonparetic limb groups.

All analyses were performed using custom MATLAB scripts (MATLAB, MathWorks, Natick, MA, USA). Individual subject data (i.e., measured across strides) and group data (i.e., healthy, non-paretic, and paretic limb data) are reported as the median and analyzed using nonparametric tests. For all analyses, α was set to 0.05.

## 3. Results

### 3.1. Stride Time Measurement Using a Thigh IMU

IMU-based measurement of stride time ($ST_{est}$) strongly approximated ground truth motion capture measurements ($ST_{act}$) for study participants from both the healthy (MAE = 7 ms, ICC = 0.986) and post-stroke (paretic limb: MAE = 15 ms, ICC = 0.996; nonparetic limb: MAE = 28 ms, ICC = 0.971) cohorts (Table 2).

### 3.2. Walking Speed and Stride Length Estimation Using a Thigh IMU

In the validation (i.e., untrained) dataset, walking speed estimates ($v_{est}$) strongly approximated ground truth motion capture measurements ($v_{act}$) for the healthy cohort (MAE = 0.035 m/s, ICC = 0.98) and for the paretic (MAE = 0.030 m/s, ICC = 0.99) and the non-paretic (MAE = 0.028 m/s, ICC = 0.99) limbs of the post-stroke cohort (see Figure 2C for both training and validation results). Similarly, stride length estimates ($SL_{est}$) strongly approximated ground truth motion capture measurements ($SL_{act}$) in the healthy cohort (MAE = 0.033 m, ICC = 0.96) and for both the paretic (MAE = 0.035 m, ICC = 0.99) and non-paretic (MAE = 0.034 m, ICC = 0.99) limbs of the post-stroke cohort (see Figure 2D for both training and validation results).

### 3.3. Distance-Induced Changes in Walking Speed and Spatiotemporal Determinants

Given highly accurate IMU estimates of walking speed, stride length, and stride time, distance-induced changes in these gait parameters can be measured using the IMU data collected during the 6-min walk test. Figure 3 and Figure 4 present the high degree of variability in these metrics that is present during long-distance walking both within and across the healthy and post-stroke cohorts.

### 3.4. Phase Portrait Roundness

**Figure 5:**
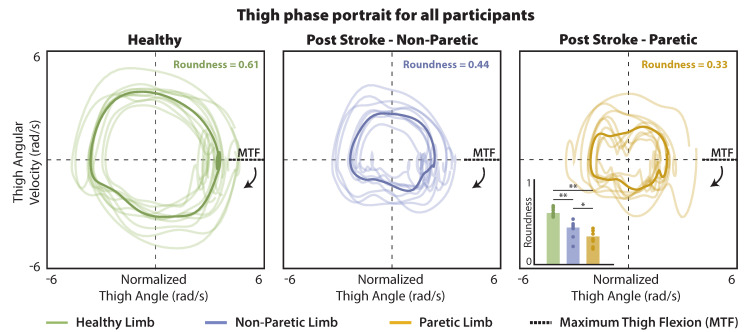
Thigh phase portraits used for speed estimation. The transparent lines in each panel are data from individual participants. The thick line in each panel is the average across participants. Subpanel: Comparison of the phase portrait roundness across groups. ** statistical significance (*p* < 0.001), * statistical significance (*p* = 0.038).

The roundness of the thigh phase portraits measured during healthy walking was significantly different from the phase portrait roundness measured for both the non-paretic and paretic limbs of the post-stroke cohort (Figure 5). Thigh phase portraits in the healthy cohort had a median roundness measurement of 0.61 ± 0.10; non-paretic limb phase portraits were 28% less round (i.e., 0.44 ± 0.10) and paretic limb phase portraits were 46% less round (i.e., 0.33 ± 0.18) ($p^{\prime }s<0.001$). Moreover, the paretic limb phase portraits were 25% less round than the non-paretic limb phase portraits (p=0.038).

## 4. Discussion

Inertial measurement units (IMUs) are portable, low in cost, and can provide real-time movement monitoring across laboratory, clinical, and free-living settings. The main finding of this paper is that a single IMU worn on the thigh can be used to produce highly accurate and robust estimates of long-distance walking speed and its spatiotemporal determinants in both healthy individuals and people with post-stroke hemiparesis. More specifically, we found that measurements of the angular position and velocity of the thigh can be taken together with the polar radius of the phase portrait produced by coupling these variables to estimate walking speed, stride time, and stride length on a stride-by-stride basis. Moreover, an exploratory analysis revealed that the roundness of the thigh phase portrait can differentiate between healthy, paretic, and non-paretic limbs, suggesting it may be a useful movement biomarker. In the near term, this work can be used in combination with our previous research advancing an IMU-based approach to estimating the anterior-posterior ground reaction forces produced by each limb during walking [17] to expand the measurement capabilities of motion capture laboratories to clinically salient, long-distance walking tests. This work also has longer-term potential to extend laboratory-grade motion analysis beyond the physical constraints of the laboratory, to the clinic and everyday world.

### 4.1. Estimating Walking Speed and Stride Length Using Thigh Phase Portraits

Across diagnostic groups, measurements of walking speed and its spatiotemporal determinants (i.e., stride time and stride length) have been strongly linked to cognition [51], fall risk [52], walking effort [4], physical activity [2,53,54], health [53,55], and mortality [55]. Wearable sensors that can continuously collect walking speed metrics in the background of everyday walking activities have the potential to inform new data-driven healthcare paradigms [11], from prevention to rehabilitation. For example, long-term tracking of these variables can identify gait changes known to precede a health decline and trigger preventative treatments that prolong health, function, and quality of life. Moreover, long-term rehabilitation efforts for chronic conditions can benefit from ecologically valid movement assessments conducted in the everyday world.

IMU-based gait analysis is an emerging subfield in biomechanics and movement science (see [11,35] for a review). Given the highly heterogeneous nature of gait deficits within and across neurological diagnostic groups, gait estimation methods developed for healthy walking need to be validated in target patient populations [11]. Recent work has shown that distally-worn IMUs (i.e., on the shank or foot) can be used to estimate stride length with a MAE as low as 0.03 m in people post-stroke [28,29]. We are not aware of speed-estimation studies conducted in people post-stroke that report stride-level error metrics (i.e., RMSE or MAE). To the best of our knowledge, this is the first paper to develop and validate an estimation approach using data from a single thigh-worn IMU.

In this paper, we describe the use of IMU-measured thigh angular position and vel-ocity to estimate healthy and post-stroke walking speed. Importantly, our sample of individuals with post-stroke hemiparesis presented with a wide range of walking speeds, ranging from 0.3 to 1.9 m/s (see Figure 2). When considering error on a stride-by-stride basis—which is an important consideration for real-time feedback and robotic control applications, and necessary for computing variability metrics—we observed highly accurate (MAE < 0.03 m/s; RMSE < 0.04 m/s) and reliable (ICC > 0.98) estimates. Moreover, for each individual, when averaging walking speed estimates across all measured strides, the average error observed was negligible (i.e., <0.005 m/s). That is, our thigh phase portrait estimation approach yields average estimates across strides that are practically equivalent to ground truth motion capture measurements.

### 4.2. Measuring Stride Time Using a Thigh IMU

Stride time is defined as the time it takes to complete one gait cycle. Using consecutively measured heel strikes to segment time-series data into individual gait cycles is the norm in the field [44]. When the detection of the heel strike is not possible, other practical or reliable gait events have been successfully used for segmentation [26,36]. Due to the cyclical nature of walking, stride times computed using different gait events should be similar. Consistent with this understanding, stride times measured between consecutive maximum thigh flexion events recorded by the thigh IMU were very similar to the stride times computed using the ground-truth measurements of heel strike (Healthy: MAE < 10 ms; Post-stroke: MAE < 30 ms, Table 2). For context, these error magnitudes, which were extracted from stride-by-stride estimates made during a non-steady-state long-distance walking test, are lower than the natural variance observed during steady-state walking in healthy individuals (i.e., between 12 and 22 ms) [56] and people post-stroke (i.e., between 50 and 130 ms) [57].

### 4.3. Distance-Induced Changes in Walking Speed and Its Spatiotemporal Determinants

Because walking speed is directly proportional to stride time and stride length, stride length estimates can be derived if walking speed and stride time are known. Based on this understanding, we were able to compute highly accurate (MAE < 0.037 m) and reliable (ICC < 0.96) estimates of stride length using our IMU-based estimates of walking speed and stride time. Building on prior work that reported the distance-induced changes in walking speed as a determinant of real-world community walking activity by people post-stroke [4], our estimation approach allows examining the interaction among walking speed, stride length, and stride time over the course of a long-distance walking test. Evaluation at this level has the potential to provide valuable biomechanical insights into the nature of an individual’s gait impairment.

For example, healthy individuals are thought to modulate walking speed and stride time in such a way that the total energy expenditure required to walk a certain distance is minimized [58,59]. More specifically, every walking speed has an individual-specific, metabolically ideal combination of stride time and stride length, with these variables modulated in a way that minimizes energy expenditure across walking speeds. However, post-stroke walking is characteristically inefficient and metabolically expensive [60,61,62], and some individuals may not be able to modulate their stride times or stride lengths to minimize energy expenditure across speeds; other factors like stability may be prioritized [63]. Instrumenting the 6-min walk test with IMUs worn on the thigh can provide insight into whether patients utilize a spatial or temporal strategy to modulate walking speed during long-distance walk tests, and ultimately direct gait therapies targeting improved walking economy and stability.

### 4.4. Phase Portrait Roundness

In an exploratory analysis, we observed that the roundness of the thigh phase portraits measured during long-distance walking could differentiate between healthy and post-stroke walking, and between the paretic and non-paretic limbs of people post-stroke. Though preliminary, this finding suggests that phase portrait roundness may be useful as a biomarker of post-stroke gait impairment. Future studies are needed to examine if roundness metrics are sensitive to stroke severity and the response to different interventions. Beyond its roundness, other quantifiable features of phase portraits may have clinical utility. For example, knowing which specific quadrants deviate from the norm may speak to different gait asymmetries and compensatory strategies. Being able to quantify post-stroke gait impairments using a single IMU worn on the thigh has the potential to substantially advance clinic-based gait analysis. Importantly, though the estimation models used in this study to estimate walking speed and, by extension, stride lengths, require subject-specific calibrations, phase-portrait roundness and other related metrics do not.

### 4.5. Limitations

There are inherent limitations to using IMU technology for human movement analysis. In addition to drift in the component signals that must be accounted for, IMUs need to be securely mounted to the body segments to avoid physical drift or movement of the sensor relative to the body segment. A current requirement of our IMU-based approach is mounting the IMUs such that one of the IMU planes corresponds with the sagittal plane of the segment. Future efforts that leverage techniques such as coordinate rotations [21,41,43,64] may enable an extension of this approach. The importance of a specific placement on the thigh segment also needs to be considered. Finally, the speed estimation equation, in its current form, requires an in-lab calibration to identify the subject-specific coefficients that relate the polar radius to speed. Future efforts that produce generalized equations would greatly expand access.

## 5. Conclusions

A single inertial sensor worn on the thigh can be used to produce highly accurate and reliable stride-by-stride estimates of walking speed and its spatiotemporal determinants during long-distance walking tests that approximate everyday community walking activities. This work has near-term potential to overcome logistical hindrances to including long-distance walking speed and spatiotemporal assessments during laboratory-based movement research and has long-term potential to facilitate new data-driven healthcare paradigms that can benefit from continuous monitoring of these salient gait parameters in free-living settings.

## Figures and Tables

**Table 1:** Study participant characteristics.

Participant Number	Side of Paresis	Stroke Onset (y)	Sex	Age (y)	Height (cm)	Weight (kg)	CWS (m/s)	6MWT Distance (m)
Healthy study participants								
H01	-	-	F	33	155	54.0	1.10	583
H02	-	-	F	25	164	46.9	1.29	602
H03	-	-	F	24	174	64.6	1.64	764
H04	-	-	M	29	179	63.9	1.19	595
H05	-	-	F	23	154	55.6	1.55	705
H06	-	-	F	25	162	64.4	1.36	651
H07	-	-	M	21	179	57.6	1.25	653
H08	-	-	M	27	179	101.2	1.22	703
H09	-	-	M	30	177	91.2	1.23	579
H10	-	-	M	25	183	78.0	1.42	834
Average	-	-	-	26 ± 4	171 ± 11	68 ± 17	1.33 ± 0.17	667 ± 85
Study participants with post-stroke hemiparesis								
S01	Left	8.08	M	61	180	72.6	0.97	495
S02	Right	5.92	M	35	184	93.0	1.47	546
S03	Left	7.92	M	78	181	100.8	1.00	314
S04	Right	7.25	M	56	180	88.0	0.80	343
S05	Left	6.08	M	62	176	99.8	1.27	516
S06	Right	3.67	M	62	176	83.0	0.70	295
S07	Left	1.75	M	67	175	87.2	0.83	303
S08	Right	2.33	M	65	171	77.1	0.39	142
Average	-	5.4 ± 2.5	-	61 ± 12	178 ± 4.1	88 ± 10	0.93 ± 0.33	369 ± 138

Abbreviations: CWS—comfortable walking speed, 6MWT—6 min walk test.

**Table 2:** Stride time measurement accuracy.

(${\mathrm{ST}}_{\mathrm{act}}-{\mathrm{ST}}_{\mathrm{est}}$)	Healthy (N = 335)	Stroke-Paretic Limb (N = 126)	Stroke-Non-Paretic Limb (N = 129)
Below −50 ms	0%	0%	7.0%
−50 to −30 ms	0.3%	4.0%	3.1%
−30 to −10 ms	25.1%	21.4%	24.0%
−10 to +10 ms	45.4%	28.6%	24.0%
+10 to +30 ms	28.7%	32.5%	29.5%
+30 to +50 ms	0.6%	10.3%	6.2%
Above +50 ms	0%	3.2%	6.2%
MAE:	7 ms	15 ms	28 ms
RMSE:	9 ms	21 ms	56 ms
ICC:	0.986	0.996	0.971

Abbreviation: HS—heel strike; MTF—maximum thigh flexion; ST—stride time; MAE—mean absolute error; RMSE—root mean square error; ICC—intraclass correlation coefficient.

## Data Availability

All data can be provided upon written request to the authors.

## Abbreviations

The following abbreviations are used in this manuscript:

IMU	inertial measurement units
6MWT	6-min walk test
MAE	mean absolute error
RMSE	root mean square error
ICC	inter-class correlation
MTF	maximum thigh flexion
SL	stride length
ST	stride time

## Appendix A. Effects of Filtering Method and Polar Radius Selection on Walking Speed Estimation Accuracy

Here we build on and validate prior work by Quintero et al. wherein it was first proposed that the polar radius of a thigh phase portrait could be used for speed estimation [26]. Their study included preliminary data for a single healthy subject walking on a treadmill. In this supplementary study, we applied their estimation approach as described (Approach A) to the healthy and post-stroke overground walking datasets collected. We compared the accuracy of Approach A to our approach that included conceptually driven changes to the polar radius values used and the filtering approach (Approach B). More specifically, as described in Section 2.4.2, to enhance estimation accuracy for people with atypical gait patterns, Approach B used the polar radius averaged across all four quadrants, rather than only the fourth quadrant as Approach A [26]. Furthermore, Approach B used a different filtering approach to better preserve kinematic deviations due to post-stroke gait deficits. Absolute and percent differences between the approaches are reported using the MAE and RMSE.

In the validation (i.e., untrained) dataset, walking speed estimates ($v_{est}$) strongly approximated ground truth motion capture measurements ($v_{act}$) for both approaches (see Table A1). Approaches A and B showed similar estimation accuracy in healthy individuals (difference = 0.002 (6%)); however, the estimation accuracy for post-stroke walking differed substantially. Approach A’s error was 37% higher for paretic limb estimates and 29% higher for nonparetic limb estimates. The increased error rate for Approach A in the post-stroke cohort is likely due to variable fourth quadrant trajectories arising from post-stroke gait deficits that are not present in healthy walking patterns. Averaging the polar radius across all quadrants (as done in Approach B) appears to reduce the effect that these deficits have on estimation accuracy.

**Table A1:** Walking speed estimation error.

	Approach A [26]	Approach B	Difference
**Healthy**			
MAE:	0.037	0.035	0.002 (6%)
RMSE:	0.047	0.046	0.001 (2%)
ICC:	0.97	0.98	-
**Stroke—Paretic**			
MAE:	0.041	0.030	0.011 (37%)
RMSE:	0.054	0.039	0.015 (38%)
ICC:	0.99	1.0	-
**Stroke—Non-Paretic**			
MAE:	0.033	0.028	0.008 (29%)
RMSE:	0.042	0.037	0.005 (14%)
ICC:	0.99	1.0	-

MAE—mean absolute error; RMSE—root mean square error; ICC—inter-class correlation.
